# Supporting data for the integrated Agent-Based Modelling and Robust Optimization on food supply network design in COVID-19 pandemic

**DOI:** 10.1016/j.dib.2022.107809

**Published:** 2022-01-10

**Authors:** Tomy Perdana, Diah Chaerani, Audi Luqmanul Hakim Achmad

**Affiliations:** a Department of Agro Socio-Economics, Faculty of Agriculture, Universitas Padjadjaran, Indonesia; b Department of Mathematics, Faculty of Mathematics and Natural Sciences, Universitas Padjadjaran, Indonesia; c Master of Mathematics Study Program, Faculty of Mathematics and Natural Sciences, Universitas Padjadjaran, Indonesia

**Keywords:** Food supply chain, Rice production, Uncertainty, Simulation, Optimization, Agent-Based Modelling, Robust Optimization

## Abstract

This article presents the data as a support for “Designing a Food Supply Chain Strategy during COVID-19 Pandemic using an Integrated Agent-Based Modelling and Robust Optimization” [1]. An integration framework of Agent-Based Modelling (ABM) and Robust Optimization (RO) is proposed to address the food supply network development involving normal and pandemic condition issue regarding the actual food production data availability. In this article, the data associated with the integrated ABM simulation and RO are discussed. Particularly, this article provides the output rice production capacity data from the ABM simulation. This article also discusses how the output data from ABM simulation are processed to construct the polyhedral uncertainty set, which will later used by RO. By showing the output data from the ABM simulation and explaining how it is processed to be used in RO, other researchers and investigators could integrate their own ABM simulation model with RO to address their respective problems considering any uncertainty. Furthermore, the additional data needed for the optimization model are also included, which are mainly retrieved from the reports of government agencies.

**Specifications Table**

**Table:** 

Subject	Management Science and Operations Research
Specific subject area	Agent-Based Modelling (ABM) and Robust Optimization (RO) for food supply network design involving normal and pandemic condition.
Type of data	Tables and figures.
How the data were acquired	The data are obtained from the ABM simulation, which gives the prediction on rice production volume given the normal and pandemic condition. Meanwhile, other input data for the optimization model are retrieved from government agencies.
Data format	Raw and analysed.
Description of data collection	There are two conditions applied within the ABM simulation: (1) normal condition and (2) pandemic condition. In other words, the impact of pandemic condition on the rice production volume is observed based on the output data from the simulation.
Data source location	• Institution: Universitas Padjadjaran • City/Town/Region: Sumedang Regency • Country: Indonesia
Data accessibility	Data are within this article
Related research article	A.L.H. Achmad, D. Chaerani, and T. Perdana. Designing a food supply chain strategy during COVID-19 pandemic using an integrated Agent-Based Modelling and Robust Optimization. Heliyon, p.e08448 (2021). https://doi.org/10.1016/j.heliyon.2021.e08448

**Value of the Data**

• These data and descriptions of how it is processed are useful to give an example of ways to integrate ABM simulation with RO to address food supply chain problems considering uncertainties.
• These data and descriptions will be useful for other researchers and investigators who would like to optimize a certain problem in their food supply chain system considering uncertainties, particularly when the required data are hard to be collected or even unavailable at the moment.
• The data processing step provides a template for organizing any uncertain data in the problem to be used in RO.

## 1 Data Description

This article supports our original research article entitled “Designing a Food Supply Chain Strategy during COVID-19 Pandemic using an Integrated Agent-Based Modelling and Robust Optimization” [1]. While our original research article provides a high-level framework for integrating ABM and RO to address uncertain food supply chain problem with unlimited data availability and its’ result, this article explains how to combine such methods with related data used. Particularly, this article explains how to integrate ABM and RO by processing the outputs from ABM to be used as the input in RO. Subsequent paragraph gives a brief explanation of ABM, RO, and why do one need to consider integrating both methods in uncertain food supply chain problem with limited data availability.

RO is a method in optimization which able to handle uncertainties in optimization problem by assuming the uncertainties are exist in a convex hull uncertainty set. Hence, it requires uncertain dataset to be used to construct its’ uncertainty set. When data availability is limited, ABM is one of the simulation methods that could be used to feed RO with the required data. ABM is chosen in our original research article as it has the unique ability to represent a system based on the actors and their behaviours, please see our original research article for high-level explanation of ABM & RO integration [1].

This section provides the output data of rice production volume from 100 repetitions of ABM simulation. The average rice production volume from 100 simulations are given in Tables 1 and 2. As reflected in Tables 1 and 2, there are two large rice production centers: Bekasi Regency and Bogor Regency. Meanwhile, the other regions are the metropolitan areas with smaller rice production capacity. The classic optimization techniques may use the average rice production volume from the 100 simulations. Nevertheless, the usage of average value is not accurate, particularly when the variations of the data are quite high. Therefore, RO is applied to handle the uncertainties of the data obtained from the ABM simulation. There are several studies incorporating types of uncertainties in the food supply chain problem as discussed by Kharisma and Perdana [3]. In this case, the uncertainties considered are the uncertain rice production volume generated from ABM simulation. The construction of polyhedral uncertainty set that gathers all the uncertainties of rice production volume is discussed in the next section, given the output data from the ABM simulation provided in this section.

**Table 1:** Average daily rice production volume (ton/day).

i-th Simulation	Bekasi City	Bogor City	Depok City	Bekasi Regency	Bogor Regency
1	48.822	40.489	32.808	286.29	1060.277
2	24.216	129.67	46.478	346.178	1240.852
3	30.985	53.769	44.525	301.132	1107.796
4	135.789	22.783	42.833	243.978	1102.979
5	120.427	80.719	49.212	214.295	917.717
6	48.561	46.218	30.985	205.962	1120.165
7	14.191	53.769	64.445	277.567	1215.074
8	128.238	51.035	100.508	366.618	879.051
9	10.025	73.818	42.833	286.29	871.369
10	33.72	162.088	40.229	264.028	1011.064
11	14.451	48.691	32.808	264.028	996.223
12	135.92	57.414	18.878	290.587	1089.439
13	25.517	2.734	47.78	289.805	954.692
14	123.942	68.35	49.212	310.506	1026.427
15	51.816	61.06	49.473	254.654	1017.964
16	0.911	28.902	122.64	323.265	1020.829
17	50.384	29.163	39.708	255.826	1055.46
18	5.859	141.518	53.769	294.883	1013.147
19	33.72	53.769	44.656	294.753	984.896
20	30.335	74.99	2.343	368.311	1265.067
21	30.985	63.663	4.557	327.04	1008.851
22	61.06	69.131	92.566	270.016	905.74
23	44.656	65.616	57.414	260.903	1006.377
24	3.645	61.06	18.357	285.9	1025.906
25	1.042	46.478	6.379	319.619	1050.773
26	46.478	35.542	9.113	262.335	935.033
27	75.771	35.542	31.246	243.327	938.027
28	33.459	25.517	158.182	352.427	950.525
29	35.542	44.656	5.598	293.581	903.787
30	18.227	57.414	25.387	269.235	878.269
31	128.238	53.769	28.251	361.671	1057.673
32	25.517	33.72	23.825	248.795	920.061
33	59.888	69.262	3.645	269.756	996.353
34	57.284	54.68	19.138	212.342	930.606
35	3.645	129.54	43.093	281.343	901.834
36	6.379	64.705	64.705	297.877	1016.663
37	38.276	57.414	55.071	244.239	891.809
38	1.562	75.641	32.938	270.667	942.323
39	44.656	30.985	54.68	282.775	1171.069
40	69.262	34.631	34.761	305.949	1173.283
41	30.465	37.365	57.414	271.839	893.762
42	37.755	38.927	91.524	283.686	960.55

**Table 2:** Continued

i-th Simulation	Bekasi City	Bogor City	Depok City	Bekasi Regency	Bogor Regency
43	44.656	51.035	33.72	259.862	978.777
44	14.581	47.39	0	199.062	655.773
45	29.944	71.345	60.278	299.7	925.008
46	81.239	151.542	49.212	241.895	1029.031
47	6.51	38.146	65.616	237.859	864.99
48	41.01	54.29	0	325.348	1050.773
49	29.163	30.985	60.148	187.996	1133.704
50	29.163	39.057	0	256.216	1154.795
51	40.099	35.412	23.695	264.809	1173.283
52	10.025	49.212	54.68	253.352	982.552
53	20.049	48.431	38.276	233.563	900.142
54	29.423	61.06	51.165	283.556	987.239
55	18.617	64.705	14.581	316.104	875.275
56	27.34	74.469	27.34	272.62	832.182
57	50.124	51.946	64.705	269.105	966.93
58	25.517	70.043	149.329	273.662	1153.624
59	46.478	120.948	64.705	281.083	973.699
60	6.379	65.616	0.911	291.107	1214.032
61	40.099	7.291	124.072	264.158	957.816
62	5.468	65.616	69.262	334.721	936.204
63	22.523	39.188	52.858	319.098	868.375
64	93.217	51.035	8.202	276.786	1089.7
65	20.961	91.785	12.759	281.864	913.421
66	41.01	48.301	20.961	243.848	1259.339
67	36.454	11.717	25.778	246.061	977.735
68	30.074	33.069	119.776	268.584	1131.621
69	12.759	6.379	17.315	245.41	812.783
70	29.163	64.705	37.365	296.706	914.983
71	161.567	61.06	58.326	258.95	829.968
72	125.374	129.15	24.606	229.787	987.109
73	163.26	69.522	0.911	274.833	1116.129
74	47.65	51.035	48.301	166.905	1123.159
75	46.478	39.448	41.14	247.363	1201.274
76	28.251	81.239	0	382.762	1026.817
77	2.734	51.946	81.5	229.657	903.917
78	0.911	73.818	0.911	253.482	922.925
79	54.42	41.922	5.468	284.207	1077.592
80	7.291	64.314	4.557	267.673	917.457
81	46.608	73.818	46.478	270.016	929.435
82	17.185	34.24	60.669	301.783	974.611
83	19.138	46.478	25.257	250.097	941.152
84	20.961	81.63	44.656	306.079	958.467

Meanwhile, other input parameters for the optimization model are given in Table 3. Monthly rice demand and rice selling price data are provided in Table 4, which obtained from West Java in Figures published by Statistics of Jawa Barat [7], [8], [9], [10], [11], [12], [13], [14], [15], [16]. Fumigation cost (Rp6.34/kg) and spraying cost (Rp7.55/kg) are considered as rice handling costs [4]. Fumigation and spraying are carried out every three months and one month, respectively. Hence, the approximation of rice handling cost in each month is Rp9,663.33/kg. For the food hub development cost, the budget estimation is Rp250,000,000/unit/month [6].

**Table 3:** Continued

i-th Simulation	Bekasi City	Bogor City	Depok City	Bekasi Regency	Bogor Regency
93	30.985	51.035	57.414	285.509	1300.349
94	14.581	54.81	0	197.5	699.647
95	102.2	61.06	42.963	275.615	744.693
96	51.946	102.2	18.227	240.593	988.411
97	71.084	51.946	1.823	254.393	1068.479
98	31.897	46.478	0	211.561	865.771
99	75.25	116.521	31.897	246.582	780.496
100	39.188	169.899	73.818	266.501	840.514

**Table 4:** Monthly rice demand and selling price.

	Bekasi City	Bogor City	Depok City	Bekasi Regency	Bogor Regency
Rice demand (ton/month)	790.486	295.723	628.294	978.951	1574.801
Rice selling price (Million Rp/ton)	11.582	11.066	11.722	11.582	11.066

## 2 Experimental Design, Materials and Methods

**Fig. 1:**
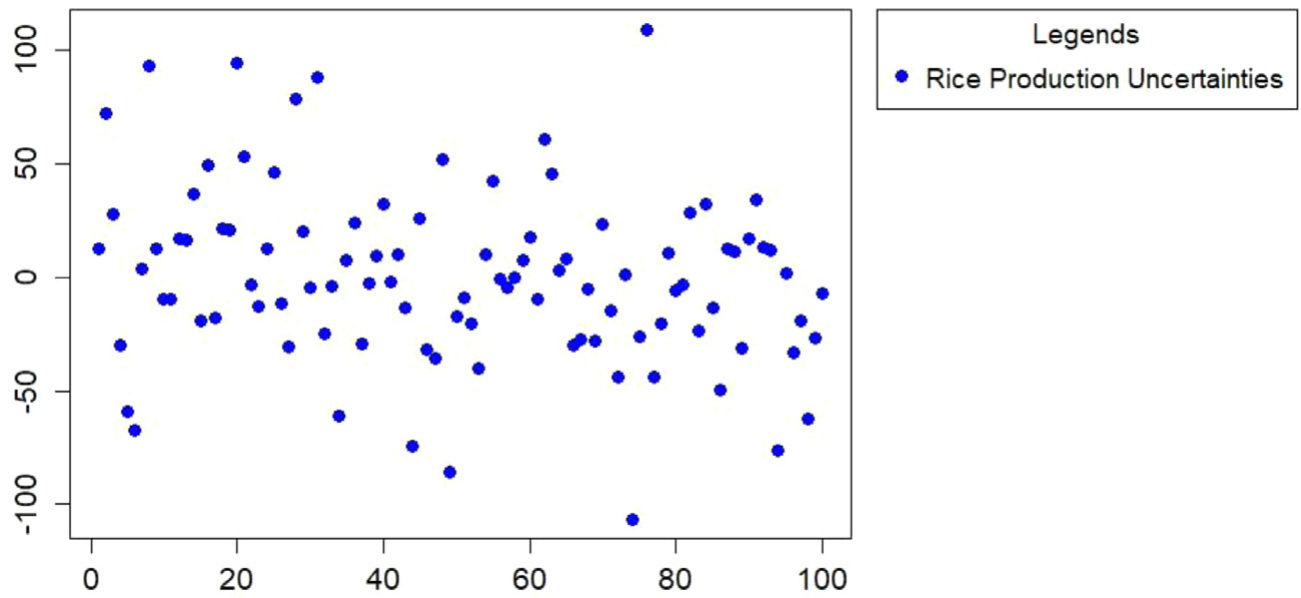
The uncertainties of daily rice production volume in Bekasi Regency [1].

This section discusses the data processing of the output data from ABM simulation to obtain the polyhedral uncertainty set. The data processing for the output data of Bekasi Regency is taken as an example. For the first step, the nominal data need to be defined, e.g., the average value of daily rice production volume in Bekasi Regency. One can calculate that the average value of daily rice production volume in Bekasi Regency is 273.628 tons/day. Once the nominal data is set, the next step is to set the uncertainty which disturbs the nominal data. In this case, the deviation of daily rice production volume becomes the uncertainty, which disrupts the nominal data as illustrated in Fig. 1. The deviation of rice production volume is given in Table 5.

**Table 5:** Daily rice production volume deviation in Bekasi Regency.

i-th Simulation	Deviation	i-th Simulation	Deviation	i-th Simulation	Deviation	i-th Simulation	Deviation
1	12.662	26	−11.293	51	−8.819	76	109.134
2	72.55	27	−30.301	52	−20.276	77	−43.971
3	27.504	28	78.799	53	−40.065	78	−20.146
4	−29.65	29	19.953	54	9.928	79	10.579
5	−59.333	30	−4.393	55	42.476	80	−5.955
6	−67.666	31	88.043	56	−1.008	81	−3.612
7	3.94	32	−24.833	57	−4.523	82	28.155
8	92.99	33	−3.872	58	0.034	83	−23.531
9	12.662	34	−61.286	59	7.455	84	32.451
10	−9.6	35	7.715	60	17.479	85	−13.636
11	−9.6	36	24.249	61	−9.47	86	−49.96
12	16.959	37	−29.389	62	61.093	87	12.793
13	16.178	38	−2.961	63	45.471	88	11.36
14	36.878	39	9.147	64	3.158	89	−31.212
15	−18.974	40	32.321	65	8.236	90	17.089
16	49.637	41	−1.789	66	−29.78	91	34.014
17	−17.802	42	10.059	67	−27.567	92	13.053
18	21.255	43	−13.766	68	−5.044	93	11.881
19	21.125	44	−74.566	69	−28.218	94	−76.128
20	94.683	45	26.072	70	23.078	95	1.987
21	53.412	46	−31.733	71	−14.678	96	−33.035
22	−3.612	47	−35.769	72	−43.841	97	−19.234
23	−12.725	48	51.72	73	1.206	98	−62.067
24	12.272	49	−85.632	74	−106.723	99	−27.046
25	45.991	50	−17.412	75	−26.265	100	−7.127

Once the nominal data and the uncertainties are defined, then one can start constructing the polyhedral uncertainty set which covers all of the uncertainties as a convex hull. In other words, one should construct the smallest possible polyhedral set which contains all of the uncertainties. There are several convex hull algorithms developed [2,5,17]. Nevertheless, the general algorithm considering n-dimensional data by taking the projections of the data points on each of 2 dimensions combination is given below:

1. Considering the n-dimensional data, take any 2 dimensions combination as the projection.
2. Based on the 2 dimensions taken, pick any single dimension as the reference.
3. Given the 2-dimensional data projections, pick a single starting point from the data which has the smallest value on the reference dimension. If there are multiple data with the smallest value on the reference dimension, then pick the data which also has the smallest value on another dimension.
4. Pick a single termination point from the data which has the biggest value on the reference dimension. If there are multiple data which have the biggest value on the reference dimension, then pick the data which also has the biggest value on another dimension.
5. Create the lower inequality constraint of the data.

5.1. Given the starting point, calculate and record the slope between the starting point and the rest of other points of data.
5.2. Based on the retrieved slopes, select another point as the endpoint which gives the smallest slopes possible with the starting point. Then, create the inequality system given the starting point and endpoint.
5.3. Set the endpoint as the starting point (move to the next selected point).
5.4. Repeat the same step from 5.1. until the starting point has reached the termination point.

6. Create the upper inequality constraint of the data.

6.1. Given the starting point, calculate and record the slope between the starting point and the rest of other points of data.
6.2. Based on the retrieved slopes, select another point as the endpoint which gives the largest slopes possible with the starting point. Then, create the inequality system given the starting point and endpoint.
6.3. Set the endpoint as the starting point (move to the next selected point).
6.4. Repeat the same step from 6.1. until the starting point has reached the termination point.

7. The polyhedral uncertainty set based on the current 2-dimensional data is obtained. Repeat the same step from 1 until all of the 2-dimensional combinations are taken.

Note that by using the above general algorithm with n-dimensional data, it is needed to define $C_{n}^{R}$ sub-polyhedral uncertainty set and combine it all together as a whole inequality system to obtain the n-dimensions polyhedral uncertainty set. In this case, the uncertain data are only 2-dimensional data. Let the $\zeta_{1}$ be the y-axis and t be the x-axis of Fig. 1. Then, the obtained polyhedral uncertainty set, which defined as an inequality system, for the uncertain daily rice production volume in Bekasi Regency is given as follows:

*ζ*1 ≤ 20*.*4400081 · *t* − 11*.*68248

*ζ*1 ≤ 1*.*3453084 · *t* + 26*.*50691

*ζ*1 ≤ −75*.*1202844 · *t* + 1097*.*02521

*ζ*1 ≥ −17*.*8361854 · *t* + 26*.*59371

**Fig. 2:**
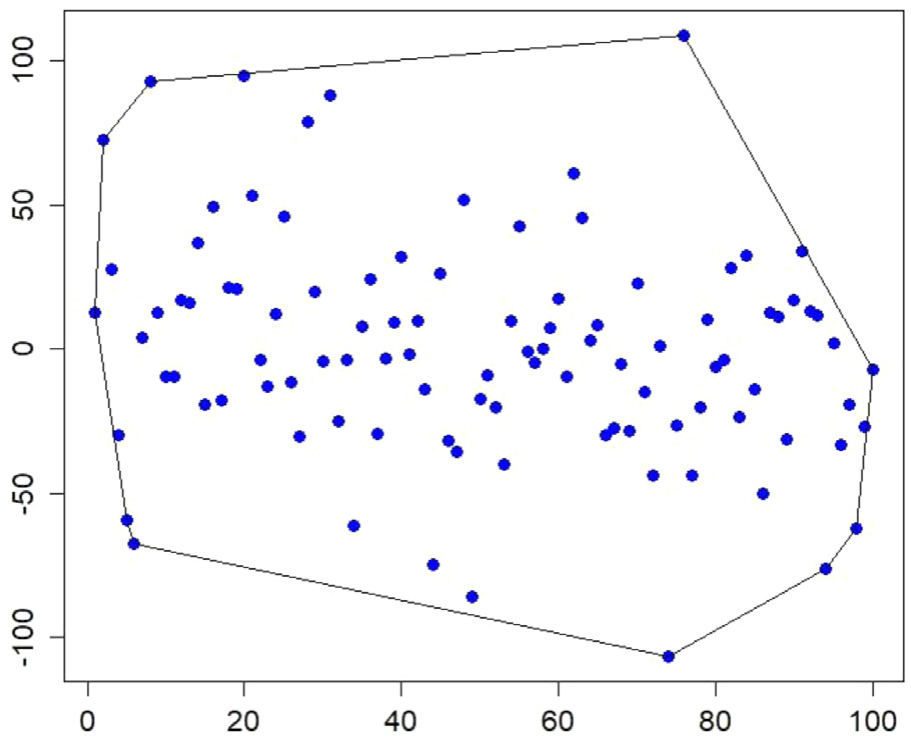
Polyhedral uncertainty set for uncertain daily rice production volume in Bekasi Regency [1].

*ζ*1 ≥ −0*.*2386837 · *t* − 26*.*19880

and illustrated in Fig. 2.

## CRediT authorship contribution statement

**Tomy Perdana:** Conceptualization, Methodology, Validation, Investigation, Resources, Writing – review & editing, Supervision, Project administration, Funding acquisition. **Diah Chaerani:** Conceptualization, Methodology, Validation, Formal analysis, Investigation, Writing – review & editing, Supervision. **Audi Luqmanul Hakim Achmad:** Conceptualization, Methodology, Software, Validation, Formal analysis, Investigation, Data curation, Writing – original draft, Writing – review & editing, Visualization.

## Declaration of Competing Interest

The authors declare that they have no known competing financial interests or personal relationships that could have appeared to influence the work reported in this paper.

## Appendix Supplementary materials

Image, application 1
